# Estimation of optimal adherence threshold for tumor necrosis factor inhibitors in rheumatoid arthritis

**DOI:** 10.1007/s10067-024-06971-y

**Published:** 2024-06-10

**Authors:** Jennifer Toth Harris, Yi Yang, John P. Bentley, Yixin Chen, Sujith Ramachandran

**Affiliations:** 1 Verana Health, 360 3rd St, San Francisco, CA 94107 USA; 2 University of Mississippi, P.O. Box 1848, University, MS 38677 USA

**Keywords:** Adherence threshold, Claims, Medicare, Proportion of days covered, Rheumatoid arthritis, Tumor necrosis factor inhibitors

## Abstract

**Introduction:**

Optimal adherence thresholds can vary across medications and disease states. The objective of the study was to determine the optimal threshold of the proportion of days covered (PDC) for tumor necrosis factor (TNF) inhibitors in patients with rheumatoid arthritis (RA).

**Methods:**

Patients with RA initiating self-administered TNF inhibitors were identified using 2012–18 Medicare fee-for-service claims. Time-varying PDC was calculated every day for the preceding 90 days during follow-up. Oral and injected glucocorticoid use, hospitalizations, emergency room (ER) visits, serious infections, and a composite of these were measured as outcomes. Time to first occurrence of each outcome as a function of time-varying PDC for TNF inhibitors was evaluated using Cox regression. Incident/dynamic time-dependent receiver operating characteristic curves and Youden’s *J* index were used to obtain the optimal PDC threshold for outcomes at 365 days.

**Results:**

Of the 1190 patients who met the study inclusion criteria, almost 75% (865 patients) experienced at least one of the outcomes. Increasing PDC by 10% was significantly associated with decreased risks of the composite outcome (HR 0.98, 95% CI 0.96–1.00), oral glucocorticoid use (HR 0.93, 95% CI 0.91–0.96), and hospitalization (HR 0.96, 95% CI 0.94–0.99) but an increased risk of ER visits (HR 1.04, 95% 1.01–1.07). Optimal PDC thresholds for the composite outcome, oral glucocorticoid use, and hospitalization were 0.64, 0.59, and 0.56, respectively.

**Conclusions:**

Increased PDC was associated with a decreased risk of adverse outcomes, except ER visits. The optimal PDC for TNF inhibitors in Medicare patients with RA based on clinical outcomes was about 60%.

**Table:** 

**Key Points** • *The optimal proportion of days covered threshold for tumor necrosis factor inhibitors at 365 days based on clinical outcomes was found to be about 60%, which is lower than the traditional 80% used to define adherence.* • *Increased adherence was associated with decreased risks of oral glucocorticoid use, hospitalization, and the composite outcome. However, it was also associated with an increased risk of emergency room visits.* • *The mean time-varying 90-day proportion of days covered decreased throughout the study starting 92% at day 1 of follow-up to 62% at day 365.*

**Supplementary Information:**

The online version contains supplementary material available at 10.1007/s10067-024-06971-y.

## Introduction

Treatment with disease-modifying antirheumatic drugs (DMARDs) is the only method for reducing inflammation and slowing joint damage caused by rheumatoid arthritis (RA) [1]. A tumor necrosis factor (TNF) inhibitor is recommended as second-line therapy in patients with moderate or high disease activity, according to the American College of Rheumatology’s 2015 treatment guidelines after the failure of monotherapy with methotrexate [2]. Medication adherence, or taking the medication at the prescriber-recommended dose, frequency, and timing [3], has been found to be associated with patient outcomes in various chronic disease states, including RA [4–7]. These studies support the positive effect of medication adherence, especially when it is dichotomized at the 80% threshold, on patient outcomes. For example, patients who self-reported taking their TNF inhibitor as directed had better responses on the 28-joint disease activity scale at 6 months [6]. In patients with various autoimmune disorders, a medication possession ratio (MPR) ≥ 80% for self-administered TNF inhibitors was associated with a 12% decreased odds of hospitalization and 18% decreased odds of an emergency room visit [7]. However, it is unknown if there are lower adherence thresholds that can yield favorable outcomes.

Currently, the 80% adherence threshold is used to dichotomize patients into adherent and non-adherent groups. This threshold was initially supported by Haynes et al., who found that patients who used at least 80% of their antihypertensive medications for 6 months had significantly lower diastolic blood pressure [8, 9]. However, the International Society for Pharmacoeconomics and Outcomes Research (ISPOR) recommends that adherence be kept as a continuous variable unless a threshold has been previously validated [10]. The 80% adherence threshold has been validated for other chronic disease states but not in RA. For example, in hypercholesterolemia, an MPR ≥ 80% to statins is associated with achieving low-density lipoprotein cholesterol goals [11]. The Pharmacy Quality Alliance (PQA) set their measure for adherence to non-infused biologics for RA to a proportion of days covered (PDC) of 80% [12]. The 80% threshold for adherence has become the norm in RA studies, cited as the value typically used [5, 13, 14]. Although the 80% threshold is normally used to categorize patients as adherent and non-adherent, the determination of this threshold in RA patients using RA-related outcomes has not been published [15].

An evidence-based threshold for adherence is needed for a couple of reasons. First, healthcare providers need a threshold for determining if non-response to medication is due to ineffectiveness of the drug or non-adherence. Patients may have their medications unnecessarily switched when increasing adherence would improve their response to the medication. The second reason is for research and quality improvement. Much current research on adherence to DMARDs for RA classifies patients as adherent or non-adherent based on the 80% threshold and relates this classification to outcomes, costs, and predictors [5, 7, 13, 16–19]. If adherence to self-injected TNF inhibitors for RA becomes a quality measure for health plans, it is important that the threshold is set using an evidence-driven approach so that outcomes are maximized if a higher threshold is needed, and providers are not unfairly penalized if a lower threshold is needed. The objective of this study was to determine the optimal threshold for adherence for TNF inhibitors with or without methotrexate among Medicare-enrolled adults with RA.

## Methods

### Study design and data source

This retrospective cohort study used the 2012–2018 5% Medicare national sample administrative claims data. The University of Mississippi Institutional Review Board (IRB protocol #22–003) approved this study, and the Centers for Medicare and Medicaid Services approved using of Medicare data (DUA#RSCH-2022–57773).

### Study sample

Patients who were diagnosed with RA and started their first TNF inhibitor therapy between July 1, 2012, and December 31, 2017, were included. Only subcutaneously administered TNF inhibitors were considered for this study as infused TNF inhibitors, which are provider administered and often have longer dosing intervals, may not be comparable to self-administered TNF inhibitors [17]. The index date was defined as the date of the first subcutaneous TNF inhibitor claim. The baseline period consisted of at least 6 continuous months before the index date, and the follow-up period lasted at least 9 months after the index date. Patients with RA were identified by the presence of two International Classification of Diseases, 9th or 10th Revision, Clinical Modification (ICD-9-CM or ICD-10-CM) codes for RA (Supplement Table 1), at least 30 days apart in the baseline period [20]. Patients must also have had at least two claims for a self-administered TNF inhibitor in the study period [20, 21]. Other inclusion criteria were continuous enrollment in Medicare Parts A, B, and D for 6 months before the index date and ≥ 19 years of age at the index date.

**Table 1:** Baseline and clinical characteristics of 1190 patients in the final sample with rheumatoid arthritis (RA) on tumor necrosis factor (TNF) inhibitors enrolled in Medicare

Characteristic	*N* (%) or mean (SD)
Age	61.3 (12.2)
Female	958 (80.5%)
Race	
White	858 (72.1%)
Black	164 (13.8%)
Other	168 (14.1%)
Index TNF inhibitor	
Adalimumab	600 (50.4%)
Etanercept	517 (43.5%)
Golimumab	29 (2.4%)
Certolizumab	44 (3.7%)
DMARD naïve	164 (13.8%)
LIS status	821 (69.0%)
Dual eligible	690 (58.0%)
CCI	2.71 (2.5)
CIRAS (RA severity)	3.76 (1.0)
Other autoimmune disease	223 (18.7%)
Cancer	302 (25.4%)
Congestive heart failure	158 (13.3%)

*CCI*, Charlson comorbidity index; *CIRAS*, the claims-based index of rheumatoid arthritis severity; *DMARD*, disease-modifying antirheumatic drug; *LIS*, low income subsidy; *RA*, rheumatoid arthritis; *SD*, standard deviation; *TNF*, tumor necrosis factor

Patients were excluded if they had claims for biologic (bDMARDs) or targeted synthetic DMARDs (tsDMARDs) in the baseline period because the use of these medications indicates a more severe disease [1]. Patients enrolled in Medicare Advantage plans at any time during the baseline period were also excluded due to potentially incomplete claims data. Patients admitted to hospice in the baseline period were also excluded due to the changes in the standards of care for hospice benefits. Eligible patients were followed from 90 days after the index date until the first occurrence of disenrollment in traditional Medicare, start of a different bDMARD or tsDMARD, admittance to hospice, death, end of the study period (December 31, 2018), or the occurrence of an outcome of interest.

### Independent variable

The key independent variable was adherence to subcutaneous TNF inhibitors. Adherence to self-administered TNF inhibitors has been previously measured similarly to adherence to oral medications. Other studies measuring adherence to self-administered TNF inhibitors used MPR, calculated as total days supply divided by the days in the treatment period [7, 18]. This study used PDC to estimate medication adherence, accounting for overlapping pharmacy claims refills. However, because medication adherence changes over time, PDC was estimated as a time-varying variable for each day of the follow-up period based on medication fill behavior in the previous 90 days. Supplement Fig. 1 shows a timeline for adherence and outcome measurement. Days supplies of medication were adjusted for overlapping days, creating a possession record for each day during follow-up, and then estimated as a percentage of the previous 90 days in which the patient had possession of the medication [10]. Using a 90-day period allowed for enough time for PDC to be calculated and lined up well with recommended follow-up intervals of every 3–6 months if patients are in remission or have low disease activity [22]. Patients who switched to another TNF inhibitor during follow-up remained in the study as switching DMARDs has been considered a continuation of the initial drug in previous research [5].

**Fig. 1:**
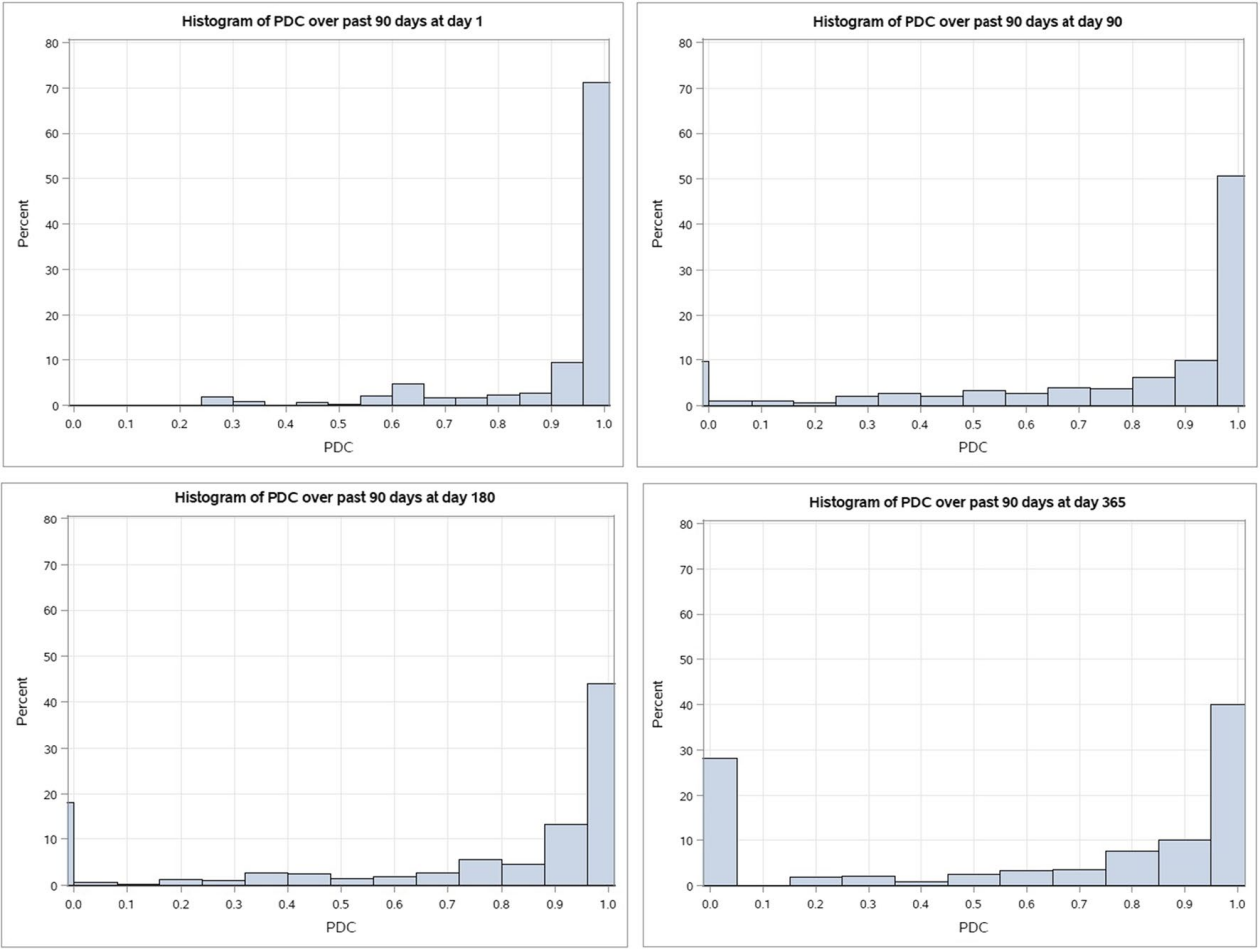
Histograms of previous 90-day TNF inhibitors PDCs at 1, 90, 180, 365 days after start of follow-up. At day 1 of follow-up, *N* = 1141, median = 1, mean = 0.924; at day 90 of follow-up, *N* = 802, median = 0.967, mean = 0.768; at day 180 of follow-up, *N* = 571, median = 0.922, mean = 0.707; at day 365 of follow-up, *N* = 330, median = 0.850, mean = 0.622. Abbreviations: PDC, proportion of days covered; TNF, tumor necrosis factor

### Dependent variables

Due to the unavailability of clinical measures like RA disease activity in claims data, proxy outcome measures were used in this study. These included the use of oral and injectable glucocorticoids (GCs), occurrence of serious infections, any-cause hospitalization, any-cause emergency room (ER) visits, and a composite outcome. Time to the occurrence of the outcomes in days from the start of follow-up, 90 days after the index date, was measured.

Oral and injected GCs were considered separate outcomes based on previous research [14]. Patients were considered injectable GC users if they received more than two GC injections on different days within any consecutive 365 days during the follow-up period [14]. Patients were considered oral GC users if they had not received oral GCs in the 6 months prior to the index date and received > 30 days of oral GCs within any consecutive 365-day period during follow-up [14]. If patients had any oral GC use in the 6 months before the index date, they were classified as oral GC users if they received > 30 cumulative days of oral GCs at doses > 120% of their average daily oral baseline GC dose [14]. Oral GC doses were converted to equivalent prednisone doses [23]. The use of injected GCs was identified based on the Healthcare Common Procedure Coding System (HCPCS), Current Procedural Terminology (CPT®), and Multum codes (Supplement Table 2). The day on which the patient met the threshold for the use of oral or injectable GCs was defined as the outcome date.

**Table 2:** Follow-up time and number of patients with RA on tumor necrosis factor inhibitors experiencing each outcome

Outcome	Patients who experienced the outcome	Median time in days after index*	IQR of follow-up time in days
Composite	865 (72.7%)	270	(165, 517)
Serious infection	191 (16.1%)	707	(407, 1239)
Hospitalization	465 (39.1%)	531	(276, 962)
ER visit	297 (25.0%)	594	(300, 1139)
Oral glucocorticoid	435 (36.6%)	517	(233, 992)
Injected glucocorticoid	247 (20.8%)	643	(360, 1174)

*Time to outcome was calculated as time from index date to first occurrence of either the outcome or a censoring event; ER, emergency room; *IQR*, interquartile range

The occurrence of serious infections was included as an outcome measure because previous literature has demonstrated such risks among patients using TNF inhibitors [24]. Serious bacterial, viral, or opportunistic infections were identified if a corresponding ICD-9-CM or ICD-10-CM code was present in the principal diagnosis field of an inpatient claim (Supplement Table 1) [25]. The presence of other outcome variables, such as hospitalizations and ER visits, was directly identified from their presence in reimbursed claims. ER visits that resulted in a hospital admission on the same day or the following day were classified as hospitalization. A composite outcome variable of all previously mentioned outcomes was also identified, corresponding to the date of occurrence of the earliest outcome.

### Covariates

Other variables of interest that may confound the key relationship of interest were classified as either time invariant or time varying. The time-varying covariates included the average weekly concomitant methotrexate dose, prescription non-steroid anti-inflammatory drug (NSAID) use, pharmacy type of dispensed TNF inhibitors (mail order/non-mail order/both), out-of-pocket pharmacy costs, and average daily number of concomitant medications [7]. Patients’ total out-of-pocket pharmacy costs during the previous 90 days were also calculated, as high out-of-pocket costs have been found to be associated with lower adherence [26]. The average daily number of concomitant medications was calculated as the total days supply from all pharmacy claims in each 90-day period divided by 90. The time-varying variables were estimated for each day of the follow-up period, similar to the estimation of PDC.

Time-invariant covariates included the index TNF inhibitor (adalimumab, etanercept, golimumab, certolizumab), age at index date, Charlson comorbidity index (CCI) based on diagnoses in the last 6 months of the baseline period [27], enrollment in the low-income subsidy (LIS) program, dual-eligible status, history of cancer, and sex. Additionally, patients were classified as DMARD naïve if they had not used baseline conventional synthetic (csDMARD) therapy, which included methotrexate, sulfasalazine, hydroxychloroquine, and leflunomide, during the baseline period. A proxy measure for disease severity was constructed at the index date using the claims-based index of rheumatoid arthritis severity (CIRAS) based on the previous 6 months of claims records [28]. The CIRAS takes into account age, sex, inflammatory tests ordered, rheumatoid factor tests ordered, number of platelet counts ordered, number of chemistry panels ordered, number of rheumatologist visits, whether there were rehabilitation visits, and whether the patient has Felty’s syndrome (Supplement Table 3). Because the following disease states use immunomodulators, the presence of any diagnosis code for plaque psoriasis, psoriatic arthritis, ankylosing spondylitis, Crohn’s disease, and ulcerative colitis in the baseline period was controlled for as the presence of concomitant autoimmune disease [18, 21, 29]. Congestive heart failure in the baseline period was also controlled for due to the potentially increased risk of hospitalization and mortality from the use of TNF inhibitors in addition to the already increased risk of cardiovascular outcomes among patients with RA [2, 30].

**Table 3:** Time-varying characteristics at 1, 90, 180, and 365 days after start of follow-up period

Characteristic	Day 1 (*N* = 1141)	Day 90 (*N* = 802)	Day 180 (*N* = 571)	Day 365 (*N* = 330)
PDC (mean, SD)	0.92 (0.16)	0.77 (0.34)	0.71 (0.39)	0.62 (0.43)
Average weekly methotrexate dose (mean, SD)	7.7 mg (10.2 mg)	7.0 mg (9.8 mg)	6.7 mg (9.8 mg)	5.9 mg (9.0 mg)
Average number of daily concomitant medications (mean, SD)	7.3 (4.4)	6.9 (4.3)	6.7 (4.0)	6.1 (3.5)
Monthly out-of-pocket pharmacy costs (mean, SD)	$99.55 ($211.34)	$65.00 ($170.41)	$60.84 ($142.06)	$82.71 ($213.46)
Pharmacy type (N, %)				
Mail order	81 (7.1%)	60 (7.5%)	46 (8.1%)	35 (10.6%)
Non-mail order	887 (77.7%)	622 (77.6%)	442 (77.4%)	241 (73.0%)
NSAID use (N, %)	305 (26.7%)	195 (24.3%)	127 (22.2%)	76 (23.0%)

Characteristics were measured over the preceding 90 days

*NSAID*, non-steroid anti-inflammatory drug; *PDC*, proportion of days covered; *SD*, standard deviation

### Statistical analysis

Descriptive statistics for baseline characteristics and time-varying variables at 1, 90, 180, and 365 days after the start of the follow-up period were calculated. Survival analysis with Cox regression was used to model time to first occurrence of each outcome using provisions for the inclusion of time-varying predictors. Data for beneficiaries who exited the study before experiencing the outcomes were censored at the time of exit. Adjusted hazard ratios (HR) and 95% confidence intervals (CIs) were calculated to estimate the adjusted association of PDC with the outcomes while holding other variables constant. PDC was rescaled to range from 1 to 10 (such that each unit represents a 10% change in PDC) to aid interpretation of the resulting HRs. As a sensitivity analysis, survival analysis models were rerun using a time-invariant PDC calculated based on the first 365 days of TNF inhibitor therapy. This sensitivity analysis only included patients who experienced the outcomes after the 365-day PDC measurement period. Those who experienced the outcomes during the 365-day PDC measurement period were excluded from the sensitivity analysis.

The optimal adherence threshold was estimated at the 365th day of follow-up to best align with the conventional approach to measuring adherence during a year of therapy. Incident/dynamic (I/D) time-dependent receiver operating characteristic (ROC) curves were created for each outcome. This use of time-dependent ROC curves allows for potentially more accurate estimation of thresholds, as it accounts for the time-varying nature of adherence as well as the changing levels of sensitivity and specificity across time [31]. The threshold with the highest Youden’s *J* index (estimated as sensitivity + specificity − 1) was selected as the optimal threshold for the outcome [32]. Area under the curve (AUC) values were calculated to determine the discriminatory ability of the model. Percentile (non-symmetric) bootstrap 95% confidence intervals were constructed for the AUCs, and *p*-values for the test that AUC = 0.5 (i.e., no discrimination) were calculated from the bootstrap samples [33]. Unadjusted and adjusted survival models were recalculated by dichotomizing PDC based on the identified threshold to estimate the impact of the dichotomized PDC on the measured outcomes.

Data management, descriptive statistics, and survival analysis were performed using SAS version 9.4 (Cary, NC), and the time-dependent ROC curve analysis was conducted using R (version 4.2.0). PROC PHREG in SAS was used to estimate survival models and hazard ratios. The R package risksetROC (version 1.0.4.1) was used to calculate the I/D ROC curves and their corresponding AUC, true positive, and false positive rates [34]. Based on existing recommendations, the local Cox method with a span equal to the number of observations in the composite set to the − 0.2 power in the survivalROC function was used to create I/D ROC curves and estimate true positive and false positive rates at each PDC value [35].

## Results

Of 13,020 beneficiaries who had a claim for a TNF inhibitor between 2012 and 2018, 1190 met the inclusion criteria (Supplement Fig. 2). Table 1 contains the baseline characteristics of the final sample. Female beneficiaries (80.5%) comprised most of the sample, and 164 (13.8%) were DMARD naïve. Most beneficiaries were enrolled due to disability benefits. Only 420 (35.3%) were enrolled in Medicare by meeting the age requirement. A total of 865 (72.7%) patients experienced at least one outcome, with hospitalizations (39.1%) being the most frequent, followed by oral GC use (36.6%), ER use (25.0%), injectable GC use (20.8%), and serious infections (16.1%). The median follow-up time, which was calculated from the index date to the first occurrence of either the outcome or a censoring event, ranged from 270 to 707 days, depending on the outcome under examination (Table 2).

Table 3 shows time-varying characteristics at days 1, 90, 180, and 365 of follow-up. The mean 90-day PDC was 0.92 (SD: 0.16) on day 1. On days 90, 180, and 365 of follow-up these numbers slightly decreased, ranging from 0.77 (SD: 0.34) at day 90 to 0.62 (SD:0.43) at day 365. Figure 1 shows histograms of the percent of patients in each PDC decile at follow-up days 1, 90, 180, and 365. Other time-varying characteristics also show a similar declining trend for average weekly methotrexate dose, NSAID use, patient out-of-pocket prescription costs, and the daily average number of concomitant medications used. The percentage of patients using only mail-order pharmacies to fill their TNF inhibitor prescriptions increased from day 1 (7.1%) to day 365 (10.6%).

Survival models using Cox regression were run for each outcome variable. Every 10% increase in PDC was statistically significantly associated with a decreased risk of the composite outcome (HR 0.98, 95% CI 0.96–1.00), oral GC use (HR 0.93, 95% CI 0.91–0.96), and hospitalizations (HR 0.96, 95% CI 0.94–0.99). The hazard ratio for PDC was significantly above 1 for ER visits, indicating that higher PDCs to TNF inhibitors are associated with a higher risk of ER visits (HR 1.04, 95%, CI 1.01–1.07). The hazard ratios for injected GCs (HR 0.99, 95% CI 0.96–1.03) and serious infections (HR 0.97, 95% CI 0.93–1.00) were not statistically significant. Table 4 shows the adjusted HRs and their confidence intervals for each variable in each survival model.

**Table 4:** Adjusted hazard ratios for each outcome in survival analysis

Variable	Composite		Serious infections		Hospitalization		ER visits		Oral GC use		Injected GC use	
	HR	95% CI	HR	95% CI	HR	95% CI	HR	95% CI	HR	95% CI	HR	95% CI
PDC^a^	0.98^*^	0.96–1.00^*^	0.97	0.93–1.00	0.96^*^	0.94–0.99^*^	1.04^*^	1.01–1.07^*^	0.93^*^	0.91–0.96^*^	0.99	0.96–1.03
Age	1.01^*^	1.00–1.02^*^	1.02^*^	1.00–1.05^*^	1.02^*^	1.01–1.04^*^	0.96^*^	0.95–0.98^*^	1.02^*^	1.00–1.03^*^	1.02^*^	1.00–1.04^*^
Male	1.06	0.88–1.26	1.15	0.81–1.63	1.19	0.95–1.50	0.67^*^	0.48–0.92^*^	0.96	0.75–1.23	1.12	0.82–1.53
Race^b^												
White	0.88	0.72–1.07	1.08	0.69–1.69	1.14	0.86–1.50	0.93	0.66–1.29	0.84	0.64–1.12	0.85	0.58–1.22
Black	0.89	0.68–1.16	1.23	0.69–2.17	1.28	0.89–1.84	0.84	0.53–1.31	0.76	0.52–1.12	0.96	0.59–1.58
Dual eligible	0.72^*^	0.58–0.89^*^	0.80	0.51–1.26	0.96	0.71–1.29	0.98	0.68–1.40	0.68^*^	0.50–0.91^*^	0.98	0.64–1.51
LIS status	1.58^*^	1.22–2.04^*^	1.50	0.88–2.56	1.44^*^	1.02–2.05^*^	1.64^*^	1.04–2.59^*^	1.09	0.77–1.54	1.03	0.63–1.68
CCI score	1.04^*^	1.01–1.07^*^	1.13^*^	1.07–1.19^*^	1.10^*^	1.06–1.14^*^	0.96	0.90–1.01	0.99	0.95–1.04	1.03	0.97–1.09
RA severity	1.13	0.99–1.28	1.16	0.89–1.52	1.13	0.95–1.34	0.71^*^	0.56–0.90^*^	1.33^*^	1.12–1.58^*^	1.20	0.96–1.52
No csDMARD use	1.19	0.97–1.45	1.46	0.99–2.16	1.31^*^	1.01–1.70^*^	1.37	0.99–1.91	1.07	0.80–1.42	1.01	0.69–1.47
No autoimmune Dx	1.06	0.88–1.28	1.12	0.76–1.64	0.91	0.71–1.15	1.00	0.74–1.36	1.30	0.99–1.71	0.71^*^	0.52–0.98^*^
No cancer Dx	0.93	0.79–1.09	1.13	0.80–1.60	0.94	0.76–1.17	0.88	0.66–1.16	1.01	0.81–1.27	0.78	0.59–1.04
No CHF Dx	0.75^*^	0.61–0.92^*^	0.57^*^	0.39–0.84^*^	0.52^*^	0.41–0.68^*^	0.89	0.60–1.32	0.86	0.65–1.16	1.50	0.99–2.28
Pharmacy type^c^												
Mail order	0.87	0.66–0.96	0.86	0.46–1.63	0.99	0.67–1.46	1.09	0.68–1.74	0.69	0.46–1.04	0.96	0.57–1.60
Both	0.97	0.80–1.17	0.65	0.42–1.01	1.06	0.82–1.34	0.77	0.55–1.09	0.94	0.71–1.23	1.39^*^	1.00–1.92^*^
Methotrexate dose	1.00	0.99–1.01	1.00	0.98–1.01	1.00	0.99–1.01	1.02^*^	1.00–1.02^*^	0.99	0.98–1.0	1.00	0.98–1.01
No NSAID use	0.83^*^	0.71–0.97^*^	0.87	0.62–1.23	0.80^*^	0.64–0.98^*^	0.76^*^	0.59–0.98^*^	1.18	0.94–1.49	0.79	0.59–1.05
Average number of daily concomitant medications	1.05^*^	1.03–1.07^*^	1.03	1.00–1.07	1.03^*^	1.01–1.06^*^	0.98	0.95–1.01	1.06^*^	1.04–1.09^*^	1.08^*^	1.04–1.11^*^
Monthly Rx Pt pay	1.00	1.00–1.00	1.00	1.00–1.00	1.00	1.00–1.00	1.00	1.00–1.00	1.00	1.00–1.00	1.00	1.00–1.00

^*^indicates *p*-value < 0.05

^a^Hazard ratios shown for PDC represent change in the risk of outcome for a 10% increase in PDC

^b^Reference category: other

^c^Reference category: non-mail order

*CCI*, Charlson comorbidity index; *CHF*, congestive heart failure; *CI*, confidence interval; *csDMARD*, conventional synthetic disease-modifying antirheumatic drug; *Dx*, diagnosis; *ER*, emergency room; *GC*, glucocorticoid; *HR*, hazard ratio; *LIS*, low income subsidy; *NSAID*, non-steroid anti-inflammatory drug; *PDC*, proportion of days covered; *Pt*, patient; *RA*, rheumatoid arthritis; *Rx*, prescription

Optimal PDC thresholds were calculated for the three outcomes, composite, oral glucocorticoid use, and hospitalizations, that demonstrated favorable outcomes at higher PDCs. The AUCs for these curves were estimated in the range of 0.5 to 0.6 and were all significantly greater than 0.5 (Table 5). The optimal thresholds for the composite outcome, oral GC use, and hospitalization were 0.64, 0.59, and 0.56, respectively (Table 5). Dichotomizing adherence at these thresholds showed a decrease in the hazard of all three outcomes in both adjusted and unadjusted models. However, the association of the dichotomized PDC was not found to be statistically significant for the composite outcome.

**Table 5:** Optimal PDC thresholds for each outcome using maximum Youden’s *J* index at 365 days

Outcome	AUC		PDC threshold	Youden’s J	Unadjusted		Adjusted ^†^	
	AUC	95% CI			HR	95% CI	HR	95% CI
Composite	0.53^*^	0.50–0.61	0.64	0.0514	0.93	0.80–1.08	0.86	0.73–1.00
Oral GC	0.53^*^	0.51–0.66	0.59	0.0491	0.61^*^	0.50–0.74^*^	0.57^*^	0.47–0.70^*^
Hospitalization	0.56^*^	0.50–0.65	0.56	0.0997	0.79^*^	0.65–0.96^*^	0.70^*^	0.57–0.86^*^

^*^indicates *p*-value < 0.05; for AUCs, *p*-values are for tests that AUC = 0.5; for HRs, *p*-values are for tests that HR = 1

^†^Adjusted HR was estimated after accounting for all covariates presented in Table 4

HR comparing PDC values above the threshold to values below the threshold. An HR < 1 indicates aPDC above the threshold has a lower risk of the outcome

*AUC*, area under the curve; *CI*, confidence interval; *GC*, glucocorticoid; *HR*, hazard ratio; *PDC*, proportion of days covered

### Sensitivity analysis

Sensitivity analysis with PDC over the first 365 days was calculated and used to determine the relationship between adherence and the outcomes using survival analysis. For this analysis, all other time-varying variables were also measured on the 365 days after the index date, and outcomes were measured any time after these 365 days. After excluding patients who experienced outcomes within the first year of follow-up, 836 patients were included in this analysis, of which 591 (70.7%), 138 (16.5%), 252 (30.1%), 343 (41.0%), 205 (24.5%), and 150 (17.9%) experienced the composite outcome, serious infection, ER visit, hospitalization, oral GC use, and injectable GC use outcomes, respectively. The average PDC over the 12 months was 0.80. PDC was not significantly related to any outcomes except for oral GC use in both simple and multivariable survival models (HR 0.89, 95% CI 0.85–0.94).

## Discussion

This is the first study to determine an optimal PDC threshold for TNF inhibitor therapy among adults with RA, which was found to be approximately 60%. Adherence to TNF inhibitors decreased as time on therapy increased. In the survival analysis, an increase in PDC was significantly associated with a decreased risk of experiencing oral GC use, hospitalization, and the composite outcome.

The PDCs found in this study for TNF inhibitors in a Medicare sample are about on par with previous studies. Calip et al. found that the mean MPR for TNF inhibitors among patients with RA was 62% over the first year [7]. Chu et al. found 1-year PDC in patients with RA who were on adalimumab or etanercept was 77% or 66% when including patients who discontinued treatment [16]. Patients with RA in a study by Grijalva et al. on adalimumab and etanercept had an MPR of 83%–85%, but those on concomitant methotrexate had lower MPRs of 72% and 64%, respectively [36]. In a study involving data from a specialty pharmacy, the mean PDC for patients on bDMARDs and tsDMARDs from index date to last fill was 86% [37]. Similarly, 2-year PDC for Optum patients with RA ranged from 81 to 87% [18]. This study found a decreasing trend in PDCs over the follow-up. While this trend may be an artifact of patients discontinuing due to being in remission or the measurement method, as there are more potential gaps in therapy over a longer duration, the low PDC values are consistent with the literature and suggest the need for continued emphasis on medication adherence among patients with RA.

Higher PDC was associated with a lower risk of the composite outcome, hospitalizations, and oral GC use, but a higher risk of ER visits. Previous studies have shown poor adherence to DMARDs usually leads to worse disease activity in RA. For example, MPR ≤ 80% to bDMARDs in the previous 180 days was associated with higher odds of a higher Disease Activity Score-28 (DAS28) score, indicating worse RA disease activity [17]. A meta-analysis found that patients adherent to DMARD therapy had lower DAS28 scores, lower erythrocyte sedimentation rates, and smaller tender joint counts, showing better adherence, which leads to improvement in these disease activity outcomes [38]. Calip et al. found having an MPR ≥ 80% was associated with an 18% lower odds of hospitalizations [7]. The increased risk of ER visits with higher PDC is surprising as it is contrary to a previous study that found an MPR ≥ 80% over 365 days was associated with a 12% lower odds of an ER visit [7]. This finding may result from confounding by indication, where patients with more severe disease are more likely to be adherent and need ER visits for pain management. While this study controlled for a claims-based disease severity score, it is possible this proxy did not eliminate this bias entirely. Interestingly, the relationship between PDC and the outcomes of interest was inconsistent in the sensitivity analysis using a time-invariant PDC measurement window. While the discrepancy in results may also be due to the low discriminatory ability of PDC, this likely demonstrates the robustness of the time-varying approach for evaluating the impact of PDC. Therefore, it should be considered the standard for future research assessing the relationships between PDC and outcomes.

The optimal PDC thresholds for oral GC use, hospitalization, and composite outcome were similar at 365 days of follow-up. This time point of interest (365 days) is in accordance with existing quality measures and other extant literature. The AUCs for these are considerably poor, although consistent with previous literature [39]. Clinical outcomes for RA—as with many other chronic diseases—are multifactorial, and adherence to medication is only one component that influences the risk of these outcomes. However, when determining a threshold for PDC, these other factors were not considered, leading to low expected AUCs. Additionally, changes in prescription fill patterns, including an increase in the use of 90-day fills and automatic refills, may lead to a departure of true medication adherence behavior from what can be captured using administrative claims data, further contributing to lower AUCs [40, 41]. The 60% adherence threshold for TNF inhibitors yielded significant adjusted HRs of less than 1 for oral GC use and hospitalization. This finding has significant implications for payers, patients, and clinicians. While this threshold is lower than what is used in existing quality measures, there may still be ample room for improvement in adherence based on the findings in this study. Although the threshold is relatively low, it is important to note that higher PDCs were associated with better outcomes. Future studies should examine the change in threshold at other times during follow-up in order to optimize potential interventions to improve medication adherence. Further research is also needed prior to changing benchmarks for quality measures—but using less stringent benchmarks provides the potential to allocate scant resources elsewhere in RA disease management. Not many studies have researched the optimal medication adherence threshold, especially in autoimmune diseases. Govani et al. determined the optimal cumulative MPR threshold for adalimumab in patients with inflammatory bowel disease was 86% and 87% for certolizumab based on a combined outcome of hospitalizations and GC prescriptions [24].

There are several limitations to the present study. Because PDC was measured based on the previous 90 days, this design potentially ignores outcomes occurring within the first 90 days after the start of TNF inhibitor therapy. Next, claims data are not collected for research purposes and, therefore, do not contain clinical information, such as RA disease activity and quality of life assessments, nor do such data capture prescriptions or medical services paid for or used outside of the Medicare program. Adherence calculations using claims data also may not reflect patients’ true medication use patterns since receipt of the medication does not indicate that the patient is actually taking the medication. Third, other patient factors and comorbidities may affect the outcomes of this study. The gold standard for measuring the clinical efficacy of TNF inhibitors is a 20% improvement in the American College of Rheumatology core set measures (ACR20) [42], which is not available in claims data. Future studies should assess the relationship between time-varying adherence to TNF inhibitors and RA disease activity. Finally, this sample of patients with RA may not represent all patients with the disease. Generalizations to other populations should be made with caution.

## Conclusion

The average 90-day PDC of self-injectable TNF inhibitors in Medicare patients with RA initially starts above 80% but then decreases to about 60% after 1 year. Higher PDC was associated with a reduced risk of hospitalization, oral GC use, and the composite outcome. The optimal PDC threshold for TNF inhibitors in RA was found to be around 60% at 365 days. More research is needed to determine the optimal adherence threshold in DMARD therapy for RA over different time intervals and at various time points after the index date than the 90-day intervals and 365 days of follow-up used in this study.

### Supplementary Information

Below is the link to the electronic supplementary material.

Supplementary file1 (JPG 96 KB)

Supplementary file2 (DOCX 39 KB)

Supplementary file3 (DOCX 23 KB)
