# Supplementary figures and images for: Estimation of optimal adherence threshold for tumor necrosis factor inhibitors in rheumatoid arthritis
Source: Clin Rheumatol. 2024 Jun 10;43(8):2435–44. doi: 10.1007/s10067-024-06971-y (PMC11269320; doi:10.1007/s10067-024-06971-y)

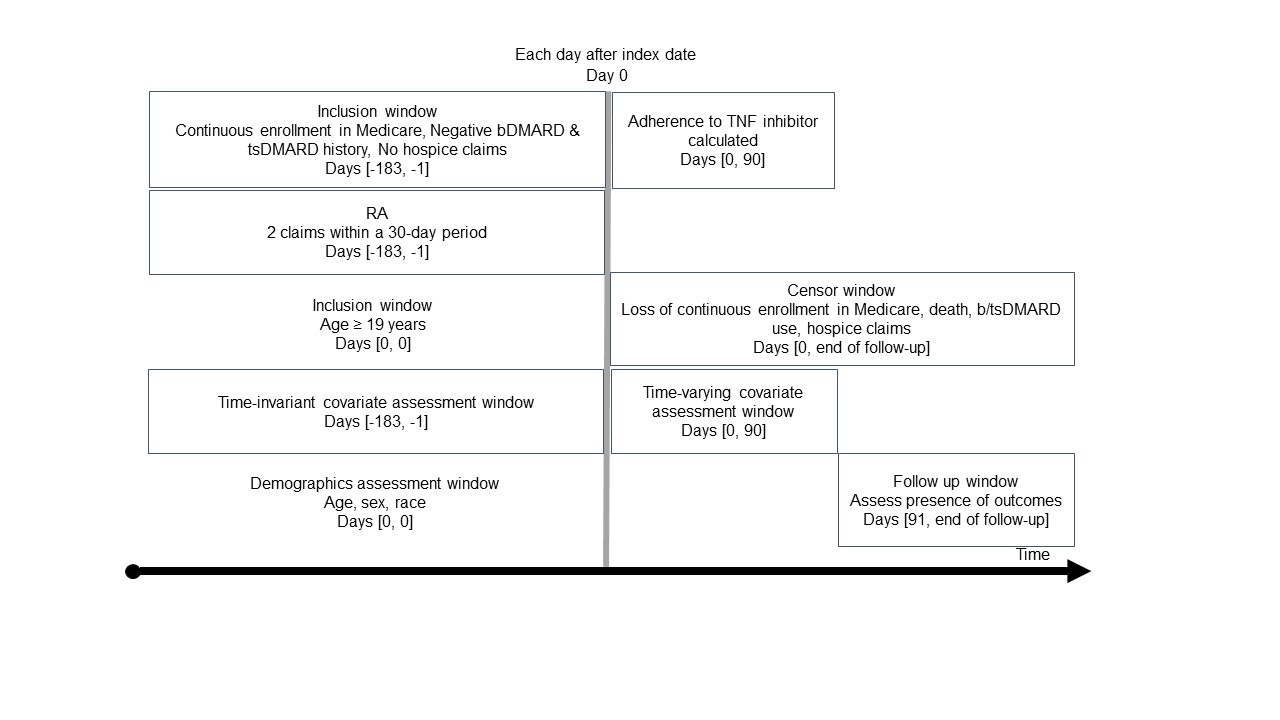

Supplement: Supplementary file 1 — Supplementary file1 (JPG 96 KB) [file 10067_2024_6971_MOESM1_ESM.jpg]
