# Supplementary material for: Estimation of optimal adherence threshold for tumor necrosis factor inhibitors in rheumatoid arthritis
Source: Clin Rheumatol. 2024 Jun 10;43(8):2435–44. doi: 10.1007/s10067-024-06971-y (PMC11269320; doi:10.1007/s10067-024-06971-y)
Supplement: Supplementary file 2 — Supplementary file2 (DOCX 39 KB) [file 10067_2024_6971_MOESM2_ESM.docx]

Beneficiaries with subcutaneous TNF inhibitor claims 2012-2018

n = 13,020

1^st^ TNF inhibitor claim outside study period or <2 subcutaneous TNF inhibitor claims

n = 5637

≥2 subcutaneous TNF inhibitor claims with first claim between July 1, 2012 and December 31, 2017

n = 7383

Not continuously enrolled 6 months prior to index date

n = 4635

Continuously enrolled in Medicare Parts A, B, D FFS 6 months prior to index date

n = 2748

<19 years

n = 0

Age ≥19 years

n = 2748

Beneficiaries without RA

n = 1254

≥2 claims >30 days apart for RA

n = 1494

bDMARD or tsDMARD claim before index date

n = 71

Received infused TNF inhibitor before index date

n = 230

First time TNF inhibitor users

n = 1423

No HCPCS codes for TNF inhibitor

n = 1193

Admitted to hospice before index date

n = 3

Final sample

n = 1190
