# Supplementary material for: Estimation of optimal adherence threshold for tumor necrosis factor inhibitors in rheumatoid arthritis
Source: Clin Rheumatol. 2024 Jun 10;43(8):2435–44. doi: 10.1007/s10067-024-06971-y (PMC11269320; doi:10.1007/s10067-024-06971-y)
Supplement: Supplementary file 3 — Supplementary file3 (DOCX 23 KB) [file 10067_2024_6971_MOESM3_ESM.docx]

Supplement Table 1. ICD-9-CM and ICD-10-CM Codes for Disease States

| **Disease** | **ICD-9-CM Code** | **ICD-10-CM Code** |
| --- | --- | --- |
| Rheumatoid Arthritis | 714.x | M05.xxx, M06.xxx |
| Psoriasis | 696.x | L40.x |
| Ankylosing Spondylitis | 720.0 | M45.x |
| Crohn’s Disease | 555.x | K50.xx |
| Ulcerative Colitis | 556.x | K51.xx |
| Cancer | 140-165, 170-172, 174-176, 179-206, 207.0x, 207.2x, 207.8x, 208.xx, 209.0x, 209.1x, 209.2x, 209.3x, 209.7x, 210.x, 211.x, 212.x, 214.8, 223.x, 225.x, 227.3, 227.4, 228.02, 230.x, 231.x, 233.7, 233.9, 235.x, 236.7, 236.9x, 237.0, 237.3, 237.5, 237.6, 237.7x, 237.9, 238.79, 239.0, 239.1, 239.4, 239.5, 239.6, 259.2, 277.89, 759.5, 759.6 | C00-C26, C30-34, C37-C41, C43.xx, C45-C49, C4A.xx, C50-C58, C60-C79, C7A.xx, C7B.xx, C80-C86, C88.2, C88.3, C88.4, C88.8, C88.9, C90-C96, D00-D03, D09.0, D09.1x, D10-D15, D17.72, D18.02, D19.0, D19.1, D20.x, D30.xx, D32.x, D33.x, D35.2, D35.3, D35.4, D37.x, D38.x, D41.xx, D42.x, D43.x, D44.3, D44.4, D44.5, D44.6, D44.7, D48.3, D48.4, D49.0, D49.1, D49.4, D49.5xx, D49.6, E34.0, K31.7, K63.5, Q85.x |
| Congestive Heart Failure | 428.xx | I50.xx |
| Serious Infections |  |  |
| Meningitis/  Encephalitis | 003.21, 036.0x, 036.1x, 049.0x, 054.3x, 062-064, 066.4x, 091.81, 094.2x, 094.81, 098.82, 320.x, 323.0x-323.4x, 323.9x | A02.21, A39.0, A39.81, A51.41, A52.13, A52.14, A54.81, A83.x, A84.x, A85.2, A87.2, A92.3x, B00.4, G00.x, G01, G04.2, G04.9x, G05.x |
| Endocarditis | 036.42, 093.2x, 098.84, 421.0x, 421.9x | A39.51, A52.03, A54.83, I33.0, I33.9 |
| Pneumonia/upper  respiratory tract  infection | 033.x, 034.x, 381.5x, 382.x, 383.0x, 383.1x, 383.9x, 461.x-463.x, 464.0x, 465.x, 466.x, 472.x, 473.x, 475.x, 480.x-488.x, 510.x | A22.1, A37.00, A37.10, A37.80, A37.9x, A38.9, A48.1, B25.0, B44.0, H66.009, H66.019, H66.13, H66.23, H66.3X9, H66.40, H66.90, H67.9, H68.009, H68.019, H68.029, H70.009, H70.019, H70.099, H70.10, H70.90, J01.00, J01.10, J01.20, J01.30, J01.40, J01.90, J02.0, J02.9, J03.00, J03.90, J04.0, J05.0, J06.x, J09.xx, J10.08, J10.1, J11.00, J11.1, J11.2, J11.81, J11.89, J12.0, J12.1, J12.2, J12.81, J12.89, J12.9, J13-J17, J18.0, J18.1, J18.9, J20.9, J21.0, J21.8, J31.x, J32.x, J36, J86.x |
| Pyelonephritis/urinary  tract infection | 590.x, 599.0x | N10, N11.0, N11.8, N12, N15.1, N15.9, N16, N28.84, N28.85, N28.86, N39.0 |
| Septic arthritis/  osteomyelitis | 003.24, 098.5x, 376.03, 711.0x, 730.0x, 730.1x, 730.2x, 730.8x | A02.24, A54.40, A54.41, A54.42, A54.49, H05.029, M00.00, M00.019, M00.029, M00.039, M00.049, M00.059, M00.069, M00.079, M00.08, M00.09, M00.10, M00.119, M00.129, M00.139, M00.149, M00.159, M00.169, M00.179, M00.18, M00.19, M00.20, M00.219, M00.229, M00.239, M00.249, M00.259, M00.269, M00.279, M00.28, M00.29, M00.80, M00.819, M00.829, M00.839, M00.849, M00.859, M00.869, M00.879, M00.88, M00.89, M00.9, M46.20, M86.10, M86.119, M86.129, M86.139, M86.149, M86.159, M86.169, M86.179, M86.18, M86.19, M86.20, M86.219, M86.229, M86.239, M86.249, M86.259, M86.269, M86.279, M86.28, M86.29, M86.60, M86.619, M86.629, M86.639, M86.642, M86.659, M86.669, M86.679, M86.68, M86.69, M86.9 |
| Gastroenteritis/  Diverticulitis | 001.x - 005.x, 008.x, 009.x, 562.11, 562.13 | A00.x, A01.00, A01.1, A01.2, A01.3, A01.4, A02.0, A02.1, A02.20, A02.22, A02.23, A02.29, A02.8, A02.9, A03.x, A04.x, A05.x,  A08.x, A09, K57.32, K57.33 |
| Viral hepatitis | 070.xx | B15.x-B19.x |
| Infectious  conjunctivitis | 032.81, 090.3x, 360.00-360.04, 372.0x | A36.86, A50.31, H10.0xx, H10.1x, H10.2xx, H10.3x, H44.0xx |
| Device associated  infections | 996.6x | T82.6XXA, T82.7XXA, T83.510A, T83.511A, T83.512A, T83.518A, T83.590A, T83.591A, T83.592A, T83.593A, T83.598A, T83.61XA, T83.62XA, T83.69XA, T84.50XA, T84.51XA, T84.52XA, T84.53XA, T84.54XA, T84.59XA, T84.60XA, T84.610A, T84.611A, T84.612A, T84.613A, T84.614A, T84.615A, T84.619A, T84.620A, T84.621A, T84.622A, T84.623A, T84.624A, T84.625A, T84.629A, T84.63XA, T84.69XA, T84.7XXA, T85.71XA, T85.72XA, T85.730A, T85.731A, T85.732A, T85.733A, T85.734A, T85.735A, T85.738A, T85.79XA |
| Skin and soft tissue  infection | 035.x, 376.01, 528.3x, 566.x, 680.x - 682.x, 684.x, 686.8x, 686.9x, 728.86, 785.4x | A46, H05.019, I96, K12.2, K61.0, K61.1, K61.3, L01.xx, L02.xx, L03.xxx,  L08.89, L08.9, M72.6 |
| Syphilis and other  venereal disease,  including  gonococcal infection | 091.x-099.x | A51-A58, A63.8, A64, M02.3xx, N34.1 |
| Tularemia/Leptospirosis | 021.x, 100.x | A21.x, A27.x |
| Opportunistic  infections | 010.x -018.x, 027.0x, 031.x, 039.x, 046.3x, 114.x-116.x, 117.3x, 117.5x, 130.x, 136.3x, 321.0x, 482.84, 484.7x | A15.x, A17.x, A18.xx, A19.x, A31.x, A32.xx, A42.0, A42.1, A42.2, A42.8x, A42.9, A43.x, A48.1, A81.2, B38.x, B39.x, B40.x, B41.x, B44.0, B44.1, B44.2, B44.7, B44.89, B44.9, B45.x, B47.1, B47.9, B48.0, B58.xx, B59, G02, H32, I32, I39, J17, L08.1, |
| Herpes zoster/simplex | 053.x, 054.x | A60.xx, B00.x, B02.x |
| Abscess | 095.2x, 324.x, 478.21, 478.22, 478.24, 540.x, 567.x, 569.5x, 572.x, 590.2x, 601.2x, 614.4x, 998.59 | A52.74, G06.x, J39.0, J39.1, K35.x, K63.0, K65.x, K67, K68.xx, K72.1x, K72.9x, K75.0, K75.1, K76.6, K76.7, N15.1, N41.2, N73.1, N73.2 |
| Septicemia/bacteremia | 036.2x, 038.x, 790.7x | A39.2, A39.3, A39.4, A40.x, A41.xx, R78.81 |
| ICD-9-CM, International Classification of Diseases, 9th Revision, Clinical Modification; ICD-10-CM, International Classification of Diseases, 10^th^ Revision, Clinical Modification | | |

Supplement Table 2. Procedure Codes and NDCs for Rheumatoid Arthritis Medications

| **Drug** | **HCPCS** | **CPT^®^** | **NDC/Multum Lexicon** |
| --- | --- | --- | --- |
| **TNF inhibitors** | **-** | **-** | 104---442 |
| Adalimumab | J0135 | 80145 | - |
| Certolizumab | C9249, J0717, J0718 | - | - |
| Etanercept | J1438 | - | - |
| Golimumab | J1602 | - | - |
| Infliximab | J1745, Q5102, Q5103, Q5104, Q5109, S9359 | 80230 | - |
| **Non-TNF inhibitor bDMARDS** | - | - | - |
| Abatacept | C9230, J0129 | - | 00003218710, 00003218713, 00003218811, 00003218821, 00003218831, 00003218841, 00003218850, 00003218851, 00003218882, 00003218890, 00003218891, 00003281411, 00003281811, 00590218710, |
| Anakinra | - | - | 55513017701, 55513017707, 55513017728, 66658023401, 66658023407, 66658023428 |
| Rituximab | C9467, J9310, J9311, J9312 | - | 50242005110, 5024205121, 50242005306, 50242010886, 50242010801, 50242010901, 64406010201 |
| Tocilizumab | C9264, J3262 | - | 50242014301, 50242014386, 50242013501, 50242013504, 50242013601, 50242013604, 50242013701, 50242013704, 50242013801, 50242013886, |
| **tsDMARDS** | - | - | - |
| Tofacitinib | - | - | 00069050114, 00069050130, 00069050230, 00069100101, 00069100102, 00069100103, 00069100201, 00069100202, 00069100203, 00069102901, 00069102902, 62157025701, 62157038301, 62157061701, 63539001202, 63539001602, 63539050114, 63539050130, 63539050230 |
| **csDMARDs** |  |  |  |
| Hydroxychloroquine |  |  | d00817 |
| Leflunomide |  |  | d04349 |
| Methotrexate |  |  | d00060 |
| Sulfasalazine |  |  | d00379 |
| **Glucocorticoids^a^** | - | 20600, 20604, 20605, 20606, 20610, 20611 | 98---301 |
| Betamethasone | J0702, J0704 | - | - |
| Cortisone | J0810 | - | - |
| Dexamethasone | J1094, J1095, J1100, J8540, S0173 | - | - |
| Hydrocortisone | J1700, J1710, J1720 | - | - |
| Methylprednisolone | J1020, J1030, J1040, J2930, J7509, K0166 | - |  |
| Prednisolone | J1690, J2640, J2650, J2920, J7506, J7510, K0167 | - | - |
| Prednisone | J1690, J2640, J2650, J7506, J7510, J7512, K0125, K0167 | - | - |
| Triamcinolone | C9469, J3300, J3301, J3302, J3303, J3304, Q9993, | - | - |
| **NSAIDs** |  |  | 58---61 |
| ^a^Intraarticular glucocorticoid injections are identified by a CPT^®^ code plus a HCPCS code  bDMARD, biologic disease-modifying antirheumatic drug; CPT^®^, Current Procedural Terminology; csDMARD, conventional synthetic disease-modifying antirheumatic drug; HCPCS, Healthcare Common Procedure Coding System; NDC, national drug code; NSAID, non-steroid anti-inflammatory drug; TNF, tumor necrosis factor; tsDMARD, targeted synthetic disease-modifying antirheumatic drug | | | |

Supplement Table 3. Scoring for rheumatoid arthritis severity CIRAS [28]

| **Criterion** | **Score** | **CPT^®^/HCPCS/ICD Code** |
| --- | --- | --- |
| Age | -0.066 | - |
| Female | -0.092 | - |
| Inflammatory Marker Test | 0.60 | 80072, 85651, 85652, 86140, 86141 |
| Rehabilitation Visits | 0.69 | 4018F, 97001, 97002, 97003, 97004, 97161, 97162, 97163, 97164, 97165, 97166, 97167, 97168, G0159, G0160, Q0086, S8990, S9129, S9131 |
| Rheumatoid Factor Test | 2.1 | 80072, 86430, 86431 |
| Felty’s Syndrome | 2.3 | 714.1, M05.0xx |
| Platelet Counts | 1 = 0.42, 2 = 0.84, 3 = 1.26, ≥4 = 1.68 | 85027, 85049, 85055 |
| Chemistry Panels | 1 = -0.14, 2 = -0.28, 3 = -0.42, 4 = -0.56, ≥5 = -0.70 | 80047, 80048, 80050, 80053, 80054 |
| Rheumatologist Visits^a^ | 0 = 0.52, 1-4 = 1.04, ≥5 = 1.56 | 99201, 99202, 99203, 99204, 99205, 99212, 99213, 99214, 99215, 99341, 99342, 99343, 99344, 99345, 99347, 99348, 99349, 99350, G0402 WITHOUT modifiers: GQ, GT, 95, POS 02 |
| Intercept | 6.5 | - |
| ^a^Also requires an RA ICD with the CPT® /HCPCS code  CIRAS, claims-based index of rheumatoid arthritis severity; CPT^®^, Current Procedural Terminology; HCPCS, Healthcare Common Procedure Coding System; ICD, International Classification of Diseases | | |
